# Isolation, Screening, and Characterization of Plant-Growth-Promoting Bacteria from Durum Wheat Rhizosphere to Improve N and P Nutrient Use Efficiency

**DOI:** 10.3390/microorganisms7110541

**Published:** 2019-11-08

**Authors:** Nilde Antonella Di Benedetto, Daniela Campaniello, Antonio Bevilacqua, Mariagrazia Pia Cataldi, Milena Sinigaglia, Zina Flagella, Maria Rosaria Corbo

**Affiliations:** Department of the Science of Agriculture, Food and Environment (SAFE), University of Foggia, 71122 Foggia, Italy; nilde.dibenedetto@unifg.it (N.A.D.B.); daniela.campaniello@unifg.it (D.C.); mariagrazia.cataldi@unifg.it (M.P.C.); milena.sinigaglia@unifg.it (M.S.)

**Keywords:** plant-growth-promoting bacteria, durum wheat, phenotypic and genotypic characterization, multivariate approach, nutrient-use efficiency

## Abstract

The main goal of this paper was to select promising microorganisms which could potentially act as plant-growth-promoting bacteria (PGPB) for durum wheat of Foggia County. At this scope, a new statistical framework, based on multivariate analyses and the evaluation of the statistical distribution of each trait, was used. Four hundred and seventy-four isolates were isolated from the rhizosphere of durum wheat in Foggia County and preliminarily screened as a function of four target indices (ammonium production, siderophores production, P-solubilization, and nitrification). After this step, the number of strains was reduced and the remaining isolates were tested through a quantitative approach, to assess the production of IAA (indole acetic acid), P-mineralization, and nitrification. In this second step, the cut-off was based on the whole population trend by evaluating for each trait the medians and quartiles. As a result, 16 promising isolates were selected and identified by 16S rDNA sequencing (*Bacillus*, *Pseudomonas*, *Stenotrophomonas*, and *Lysinibacillus*). The last step of this research was a preliminary validation in a growth chamber on eight strains. As screening and simple indices, two quantitative measures were chosen. The main result was the selection of at least three isolates (6P, 20P, and 25A) for a future field validation. They increased biomass and height by respectively 50% and 25%.

## 1. Introduction

A projection of world population of 9.1 billion and an increase of 70% of global demand for major grain crops within 2050 was reported by FAO (Food and Agriculture Organization of the United Nations) [1]. To meet this target, cereal production must increase, with a focus on sustainability. The use of bio-resources such as PGPB (plant-growth-promoting bacteria) to enhance plant growth and biocontrol seems to be a new agricultural-engineering technique to produce wheat and seems to be very attractive for numerous researchers [2].

The issue of production sustainability is even more acute in semiarid and arid regions, such as Mediterranean arable lands, where drought and related biophysical factors create a fragile and unstable environment for production [3]. In these areas, durum wheat (*Triticum turgidum* L. subsp. *durum*) is the most extensively cultivated cereal. In the past decades, agricultural practices have focused on maximizing yields by increasing fertilization, mainly N and P fertilizations. However, an excessive use of these compounds causes leaching, pollution of water resources, and gaseous emissions to the atmosphere, with irreparable consequences to the environment and human health [4]. PGPB can exert beneficial effects on plant growth, with direct/indirect mechanisms, to produce durum wheat of both sufficient quantity and quality [5,6]. Numerous reviews focused on these mechanisms and how they help enhance the performances of the plants [5,7,8,9]. Several soil microbial species exhibit P-solubilization capacity, playing an important role in the whole soil P cycle. The main P-solubilization mechanisms employed by soil microorganisms include release of complexing or mineral-dissolving compounds, e.g., organic acid anions, siderophores, protons, and hydroxyl ions; liberation of extracellular enzymes (biochemical P-mineralization); and the release of P during substrate degradation (biological P-mineralization) [10]. “Phosphate-solubilization” and “phosphate-mineralization” may coexist in the same bacterial strain.

Other effects of PGPB include their ability to enhance N availability in the rhizosphere (N-fixing bacteria and nitrifying bacteria); the increase of root length and density (i.e., indole acetic acid producer bacteria); the impact on systemic plant metabolism; and microbial phytoprotection (i.e., siderophores producer bacteria).

The isolation of PGPB and the selection of the best-performing isolates is a challenge for at least two reasons: the protocol for the isolation/selection and the management of a large amount of data.

There are many studies dealing with the isolation and the characterization of PGPB from the rhizosphere of a wide variety of crops. Generally, two possible approaches could be used. The first one is a culture-dependent method, based on the isolation of a high number of isolates, then followed by characterization and identification, and finally by the selection of the most promising candidates for field application. This approach is a step-by-step method with different phases and at least three different levels of investigation (isolate study in laboratory media, first validation in pots, and second validation in real conditions) [11]. The main result of this method is the selection of some performing strains with desired traits and properties. Generally, this method does not take into account the quantitative distribution of microbiota in the soil and the colonization (i.e., the relative abundance of each isolate); however, the main benefit is the possibility of assessing the technological robustness of the strains and including it in the selection isolates with a low abundance but interesting in terms of application and improvement of crop. Another approach was proposed by Yan et al. [12]. It is based on a preliminary metagenomic analysis, with a focus on the population diversity and on the phylogenetic tree of each family/group. The main benefit of this approach is the possibility of studying the abundance of each strain and taking into account the actual colonization in soil and on the plant. After this step, the selection is done on few isolates of each cluster. However, this approach could suffer a drawback, as isolates belonging to the same cluster could experience different phenotypic traits [13,14]. The choice of the approach (culture-dependent and isolate characterization or metagenomic approach + characterization) relies on the goal of the research. In this paper, the main goal was to isolate and select performing bacteria that were able to act as biofertilizers for durum wheat; therefore, it was important to study the traits of each isolate.

A second challenge was the management of a large amount of data. Generally, the selection of a performing microorganism is based on qualitative and quantitative analyses; after the assessment of the variables under investigation, many researchers proposed a final multivariate statistic (for example, principal component analysis) to select the best strains [15]. The choice of the clustering method is a challenge because, if the amalgamation is wrong, the use of variables with different units or that are not homogeneous (qualitative and quantitative) could lead to bias and statistic artefacts [16,17,18]. A new approach was proposed by Corbo et al. [19] to study the lactic population of a meat matrix and select some performing strains. This method is based on the assumption that each parameter should be studied alone and only in second phase studied in combination with other variables. Another assumption is that it is not possible to set an a priori cut-off; the trend of the whole population should be considered.

The main goal of this paper is the isolation, characterization, and selection of performing strains to be used as potential biofertilizer for durum wheat of Foggia County, with a special focus on the nutrient-use efficiency (N and P); nevertheless, the isolation was performed from the rhizosphere of the crop, as it is well-known that wild strains naturally possess some mechanisms of adaptive evolution and are able to win and overcome stressful conditions [19].

In addition, a goal was the optimization of step-by-step methodological approaches based on different tools and protocols, to manage a large amount of data and take into account the traits of the population.

## 2. Materials and Methods

### 2.1. Soil Sampling

Soil samples, from rhizosphere of durum wheat, were collected by grubbing up plants (cv Saragolla) grown in a farm nearby the University of Foggia (Apulia-Southern Italy 41°19′17″N 15°42′10″E) during the 2015 crop season. The samples were taken around the roots (ca. 4 cm), in order to analyze and then select bacteria from rhizosphere (Supplementary Figure S1).

Bacterial isolation was performed on a silty-clay-loam soil, with 1.3% total nitrogen content (Kjeldhal method), 34 ppm assimilable phosphorus [20], 41.4 mg/Kg organic matter (Walkley-Black method), and an 8.1 soil pH. The microbiological analyses were carried out in duplicate during waxy stage of the durum wheat life cycle.

### 2.2. Isolation of Bacteria

Aliquots of 10 g of soil were diluted with 90 mL of a sterile saline solution (0.9% NaCl solution), homogenized in a Stomacher bag (Seward, London, England), and blended for 1 min in a Stomacher Lab Blender 400 (Seward). Then, serial dilutions were carried out and plated onto appropriate medium, to select and count Mesophilic bacteria (Nutrient agar; 30 °C for 48 h), pseudomonads (Pseudomonas Agar Base added with Pseudomonas Selective Supplement; 25 °C for 48–72 h), spore-formers (PCA, after heat-treating the dilutions at 80 °C for 10 min; the plates were incubated at 30 °C for 24 h), Actinobacteria (Bacteriological Peptone, 10 g/L; Beef Extract, 5 g/L; NaCl, 5 g/L; Glycerol, 10 g/L; Agar Technical n. 3, 20 g/L; pH7.00–7.20; 22–24 °C for 7–14 days), total and fecal coliform (Violet Red Bile Agar, incubated at 37 and 44 °C for 18–24 h). All media and supplements were from Oxoid (Milan, Italy).

The measurement of pH on the homogenized product was performed twice on two different batches by using a Crison pH-meter, model 2001 (Crison Instruments, Barcelona, Spain), calibrated with two standard solutions buffered at pH = 4.00 and 7.02.

From each plate, 5 to 10 colonies with different morphology were randomly selected, isolated, purified, labelled with a numeric code, and stored at 4 °C.

### 2.3. Screening

All isolates were morphologically and biochemically characterized through phenotypic tests (Gram staining, catalase, oxidase, urease test, microscopic observation, spore production, and motility) [21].

### 2.4. Ammonium Production

The experiment was done as reported previously [22]. Bacteria were inoculated in test tubes containing Peptone Water medium [23]. The tubes were incubated at the optimal temperatures for 48–72 h (30 °C for mesophilic and spore-forming bacteria, 22 and 25 °C for actinobacteria and pseudomonads). The accumulation of ammonia was detected by the addition of Nessler’s reagent to each tube. A tenuous-yellow or a deep yellow-to-brownish color indicated a small (+) or a high (++) production of ammonium, respectively.

### 2.5. Siderophores Production

Bacteria were inoculated onto Chrome Azurol S (CAS) agar and incubated for 24 h, at their optimal temperatures [24]. An indication of siderophore production was the changed color from blue to purple (as described in the traditional CAS assay for siderophores of the catechol type) or from blue to orange (as reported for microorganisms that produce hydroxamates) halo around the colonies.

### 2.6. Nitrification

The protocol was slightly modified by Chatterjee et al. [21]. The nitrifying bacteria were enumerated on Winogradsky’s medium. The colonies were visualized (pink color) by flooding the plates with sulfanilic acid reagent.

### 2.7. Phosphate-Solubilization

Inorganic phosphate solubilizing bacteria were analyzed on Pikovasky medium, with a protocol modified by Dawwam et al. [23]. A clear zone around colonies showed the phosphate-solubilizing ability of isolates. The halo was measured in three directions, and the average was calculated. The analyses were carried out in triplicate.

### 2.8. First Selection

The analyses were carried out on three independent batches. For each experiment, two technical replications were performed. The results of the qualitative analyses (siderophores, nitrite and nitrate, phosphate-solubilization, and NH_4_^+^ production) were converted in numeric codes (0, negative in all replicates of each isolate or positive only in a replicate; 1, positive in all the replicates) and used as input values to run a principal component analysis; the Euclidean distance was used as the amalgamation method.

### 2.9. Indole Acetic Acid

The production was assayed through a colorimetric method described by Dawwam et al. [23]. For each isolate, 250 μL of cell suspension was inoculated in 5 mL of nutrient broth with tryptophan (0.1 g/L) and incubated at 30 °C for 7 days. The cultures were centrifuged (10,000 rpm for 10 min), and then 500 μL of supernatant was added, with 1 mL of Salkowski’s reagent [23] and a drop of orthophosphoric acid (85%), and incubated at room temperature for 15 min, until pink (an indicator of indole production). The quantity of indole was measured at 530 nm, using a microplate reader CLARIOstar (BMG Labtech, Ortenberg, Germany). A standard solution of pure indole-3-acetic acid was used to build a calibration curve (1–25 µg/mL pure IAA).

### 2.10. Sequential Determination of NO_2_^−^ and NO_3_^−^

To evaluate NO_2_^−^ concentration, each isolate (10^8^ CFU/mL) was inoculated in Nutrient Broth (Oxoid, Milan, Italy) and opportunely incubated. The cultures and reagents were prepared as described in García-Robledo et al. [24]. Absorbance was measured at 540 nm, using a microplate reader CLARIOstar (BMG Labtech, Ortenberg Germany). A calibration curve was built with NO_2_^−^ standards.

To determine NO_3_^−^, 650 μL of the samples previously prepared was added with 65 μL of VCl3 and analyzed as reported by García-Robledo et al. [24].

The absorbance was measured at 540 nm. A calibration curve was built with NO_3_^−^ standards.

### 2.11. Phosphate-Mineralization for Alkaline Substrate

The sodium bicarbonate (NaHCO_3_) procedure of Olsen et al. [20], which is considered to be a suitable index of P “availability”, was adapted to microbial culture [25].

The absorbance of blank (KH_2_PO_4_) and samples were read into a microplate reader, CLARIOstar BMG Labtech, at 882 nm.

### 2.12. Second Selection

The results from the quantitative analyses were analyzed by the coefficient of variation (CV), median, and quartiles. First, the replicates for each parameter and each isolate were analyzed by means of CV; if CV was > 10%, the outlier test was done to remove the replicate different from the other two. Then, the median and the quartiles of each parameter were evaluated by using all replicates of all isolates, and the quantitative data of each isolate were converted in a qualitative variable with four possible levels (0, 1, 2, and 3) (X is the assayed parameter):

Code 0, negative to the assay;

Code 1, X < median;

Code 2, median < X < 3rd quartile;

Code 3, X > 3rd quartile.

The selection criterion to choose an isolate for validation was that the assayed parameter should be at least as high as the third quartile (coded level 3). In addition, the number of the isolates was further reduced by a second selection and by choosing the isolates with the highest level of the parameter by means of one-way analysis of variance ANOVA and Tukey’s test (*p* < 0.05).

### 2.13. Identification of Isolates

The isolates were identified by sequencing the 16S rDNA. DNA was extracted via a NucleoSpin^®^ 8 Food kit from MACHEREY-NAGEL GmbH (Düren, Germany). The amplification was done in a volume of 12 µL, comprising 2 µL of DNA and 10 µL of reaction mixture. The primers16S rDNA univ 27F were used at 5 pmol (Available online: http://themicrobiome.com/en/16s/16s-primers#.Wwam0GdG270:) (Eurofins Genomics GmbH, Ebersberg, Germany). The PCR program was set as follows: initial denaturation at 95 °C for 1 min, 35 cycles of denaturation (95 °C for 15 s for each cycle), annealing (55 °C for 15 s), and extension (72 °C for 10 s) (GeneAmp™ PCR System 9700, Applied Biosystems, Foster City, CA, USA).

The purification of PCR products was done in a volume of 11 µL (1 µL of DNA and primers 27F and 1492R at 5 pmol) through the ExoSAP-IT™ PCR Product Cleanup Reagent from ThermoFisher Scientific Inc in a BigDye™ Terminator v3.1 Cycle Sequencing Kit (initial denaturation at 95 °C for 1 min, 35 cycles of denaturation at 96 °C for 15 s, annealing at 55 °C for 5 s, and extension at 60 °C for 4 min) (GeneAmp™ PCR System 9700, Applied Biosystems).

The products were analyzed on an Applied Biosystems™ 3730 DNA Analyzer (ThermoFisher Scientific Inc., Whaltham, MA, USA) with Data Collection 4.0, using the POP7 polymer mix and 50 cm Array by the sequencing department of Eurofins Genomics GmbH. The assembled sequences were compared with the sequences available in the GenBank database.

On the strains selected for 16S r-DNA sequencing, the effect on P was confirmed by studying P-solubilization in the presence of AlPO_4_ and Fe(PO_4_)_3_, as reported by Sungthongwises et al. [25].

### 2.14. Growth Chamber

#### 2.14.1. Preparation of Bacterial Inocula

The isolates 12A, 25A, 36M, 40M, 97M, 6P, 20P, and 23P were grown up to the stationary phase (10^8^ CFU/mL). In particular, 12A and 25A were incubated in Actinobacteria broth at 22–24 °C for 7–14 days; 36M, 40M, and 97M were incubated in Nutrient broth at 30 °C for 48 h; 6P, 20P, and 23P were incubated in Pseudomonas broth added to Pseudomonas Selective Supplement, at 25 °C for 48–72 h. Afterward the cultures were centrifuged at 4000× *g* for 5 min, and then they were washed twice with sterile phosphate buffer-PBS (1.24 g K_2_HPO_4_, 0.39 g KH_2_PO_4_ and 8.80 g NaCl per liter); the supernatant was discarded, and the pellet was resuspended in PBS. The viable count of the suspension was ca. 10^8^ CFU/mL. The inoculated buffer was used to inoculate *Triticum durum* seeds.

#### 2.14.2. Preparation of Seeds

Sterilization of *Triticum durum* seeds (cultivar Saragolla, durum wheat cultivar, characterized by high nitrogen-use efficiency) was performed by a dipping in 70% ethanol for 3 min, and a second dipping in a 3% hypochlorite solution for 10 min. Then, the seeds were washed with sterile distilled water and germinated on moist, sterile filter paper, in petri dishes, for 48 h.

Five seeds were treated with 1 mL of pre-inoculated buffer for 1 h; seeds in uninoculated sterile buffer represent the control [26]. The seeds inoculated and uninoculated were sown in pots under controlled conditions (Table 1).

#### 2.14.3. Growth-Chamber Assay

Saragolla was grown in not-sterile soil, under controlled conditions (Table 2), in pots (15 × 15 × 20, 3.6 L, 0.0225 m^2^). Nitrogen was applied as urea (50 kg/ha), 50% as base fertilization and 50% as cover fertilization. Phosphorous was supplied at sowing as triple superphosphate P_2_O_5_ (45 Kg/ha). Irrigation was performed with tap water, to keep optimal soil moisture (80% of available water) for wheat growth (field capacity 32%; wilting point 15%).

For each experiment, a randomized design was used, with three biological replications. In the first experiment, sowing was performed on 21 September 2017 and harvest on 13 November 2017, at tillering. Plant aerial height was determined by excluding roots, and biomass dry matter was determined after drying in a forced-air oven at 60 °C for 48 h. Microbiological analyses and determination of pH of the soil were performed during the growth cycle, as reported above.

The results of the growth chamber were analyzed via a one-way ANOVA and Tukey’s test (*p* < 0.01). Statistics were done through the software Statistica for Windows, version 12.0 (Statsoft, Tulsa, Okhla).

## 3. Results

The selection of isolates which could potentially be PGPB was performed by using three steps: in the first one, the isolates were screened for some simple qualitative indices to reduce their number and exclude the microorganisms negative to the tests; in the second step, three indices were assayed and studied to select promising strains. These strains were identified through 16S rDNA sequencing and after a third reduction of the number of isolates used for a preliminary validation in a growth chamber.

### 3.1. Qualitative Screening: First Selection

Mesophilic, and spore-forming bacteria showed the highest cell number (ca. 7 log CFU/g), whereas actinobacteria and pseudomonads were at lower levels (ca. 6 log CFU/g). Coliforms were always below the detection limit (ca. 2 log CFU/g); pH was alkaline (about 7.8) (data not shown).

Table 3 reports the results for the preliminary characterization of the isolates. As expected, the spore-forming bacteria and pseudomonads were Gram-positive and Gram-negative, respectively; in addition, Gram-positive bacteria represented the most of the population of presumptive mesophiles and actinobacteria. A positive response to oxidase was mainly found among pseudomonads, as one could expect from their oxidative metabolism; on the other hand, the output was isolate-dependent for the other groups. Concerning the response to H_2_O_2_, the isolates were mainly catalase-positive, and this trait suggested their aerobic or aero-tolerant metabolism. Other traits assessed throughout the screening were urease production and the motility at their optimal temperature of growth. Concerning urease, all spore-forming bacteria were negative to this assay; moreover, it was only found in a few isolates of the other groups. The target microorganisms were also negative to motility tests, as it was found in a low number of isolates (from 6% for actinobacteria to 30% for pseudomonads).

**Figure 1:**
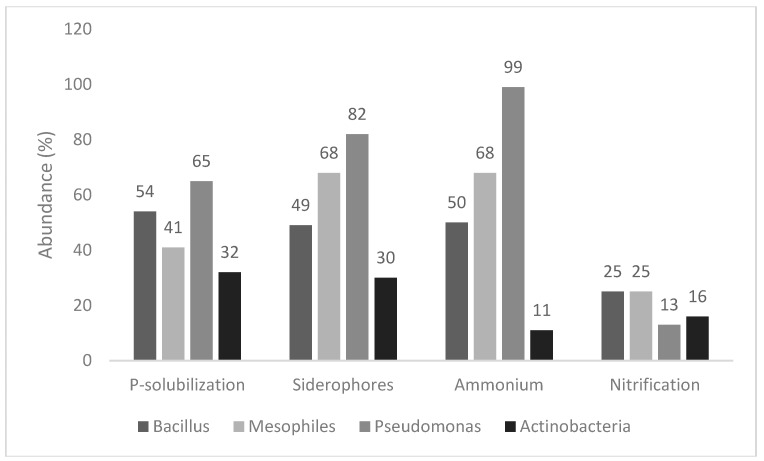
Strains positive to P-solubilization, siderophores’ production, ammonium production, and nitrification.

Figure 1 shows the response to four qualitative tests (P-solubilization, siderophores’ production, ammonium production, and nitrification), used as screening indices to point out isolates which could potentially act as PGPB. Concerning P-solubilization, the number of positive isolates was from 32% (actinobacteria) to 65% (pseudomonads), whereas mesophiles and spore-forming bacteria showed an intermediate trend (positive isolates at 41%–54%). A similar trend (the highest number of positive isolates in *Pseudomonas* spp. at 82%, and the lowest for actinobacteria 30% with mesophiles and spore-forming bacteria at 49%–68%) was recovered for siderophores.

NH_4_^+^ production was found for all pseudomonads (99%), with few exceptions, and in a low number of isolates of actinobacteria (11%), whereas the outputs were variable for mesophiles and spore-forming bacteria, as this trait was found in 50% of mesophiles and 68% of spore-formers. Finally, the 4 groups were generally negative to nitrification, as this property was only found in 13%–25% of the isolates.

The selection of a promising microorganism is a kind of a risk–benefit analysis and requires a focus at isolate-level, to analyze the outputs of each microorganism and select the most promising ones. At this scope, a multivariate analysis (principal component analysis, PCA) was used as a tool to gain insight into the complexity of the four sub-populations and reduce the number of the isolates for the second step (quantitative assessment of some selected traits). The results of the screening indices were converted to a binary code (0, isolate negative to the test; 1 isolate positive to test), to run PCA. This approach resulted in a main output: strain clustering and pointing out homogeneous groups, including the isolates with the same responses to all tests. Therefore, each figure contains two parts: strain distribution in the factorial space and a table with all isolates included in each group.

**Figure 2:**
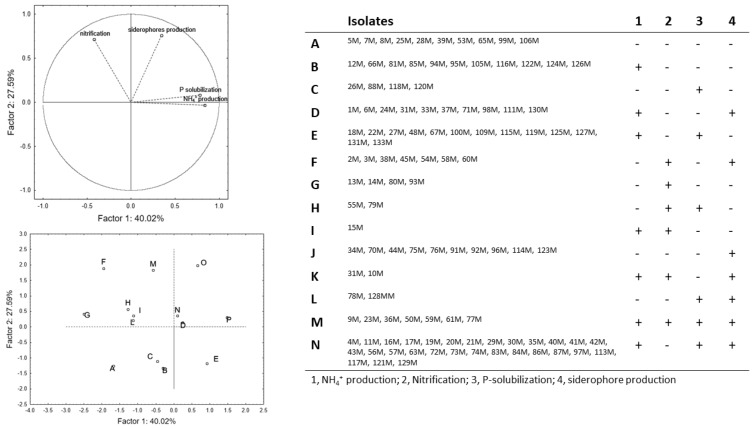
Principal component analysis run on the output to NH_4_^+^ production, nitrification, P-solubilization, and siderophores production for the mesophilic bacteria.

Figure 2 shows variable and case distribution for mesophilic bacteria; the analysis accounted for 67% of the total variance. The first factor was positively related to NH_4_^+^ production and P-solubilization (correlation coefficients of 0.834 and 0.782, respectively), while the second factor was positively related to nitrification (0.717) and siderophores’ production (0.762). The isolates could be divided in some homogeneous groups, as a function of the qualitative response to the different tests. In the quadrants I and IV, there are the isolates positive to P-solubilization and NH_4_^+^-production, whereas the microorganisms positive to siderophore and nitrification are in the quadrants I and II. The isolates in the group O (9M, 23M, 36M, 50M, 59M, 61M, and 77M) were positive to all the tests; on the other hand, the group A was composed by isolates negative to all the assays (5M, 7M, 8M, 25M, 28M, 39M, 53M, 65M, 99M, and 106M). The other groups were characterized by responses positive/negative to the different assays.

**Figure 3:**
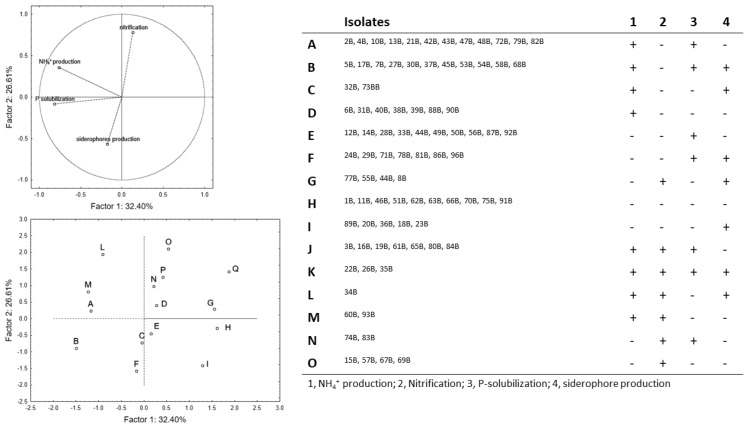
Principal component analysis run on spore-forming bacteria.

The PCA for spore-forming bacteria (Figure 3) accounted for ca. 59% of the total variability; P-solubilization and NH_4_^+^ production showed a negative correlation with the factor 1 (coefficients of −0.762 and −0.816), whereas nitrification and siderophores’ production were, respectively, related to factor 2 with a positive (0.780) and a negative (−0.568) coefficient. The group H included microorganisms negative to all assays (1B, 11B, 46B, 51B, 62B, 63B, 66B, 70B, 75B, and 91B), whereas the isolates positive to all assays are in the group M (22B, 26B, and 35B). Pseudomonads were positive to at least one trait and were divided in seven phenotypic groups (Supplementary Figure S2), but only the isolates of the group C (19P, 20P, and 30P) were positive to all assays. Actinobacteria showed a high level of complexity, with 15 different phenotypic groups (Supplementary Figure S3).

Generally, PCA suggested a high level of biodiversity and complexity, thus pointing out the need of more restrictive inclusion criteria. Therefore, the viability/robustness was chosen as the main requisite, and the isolates showing a viability loss throughout storage were excluded.

Moreover, while all remaining strains were studied for IAA (indole acetic acid) production, only the isolates positive to P-solubilization and nitrification were assayed for the quantitative determination on P-mineralization and sequential production of nitrites and nitrates. As a result, the number of experiments was reduced from 1422 (isolates x 3 assays) to 333 (206 isolates to be tested for IAA, 82 for P-solubilization, and 45 for nitrification).

### 3.2. Quantitative Analyses and Identification: Second Selection

The data from the quantitative assays were analyzed by some simple descriptive indices (median and quartiles), to study the statistical distribution of each parameter within each microbial group. The results are in Table 4. The main criterion to select an isolate was that the quantitative index (P-mineralization, nitrification, and IAA production) should be at least as high as the third quartile (coded level 3) (Supplementary Table S1). In addition, the number of the isolates was further reduced by choosing the isolates with the highest level of the parameter by means of one-way ANOVA homogeneous-group approach.

As an example, Supplementary Table S2 reports one-way ANOVA/method of homogeneous groups for P-mineralization. By using the tables of the homogeneous groups, 15 isolates were selected (six in the group of the mesophilic bacteria, four among spore-forming, one for pseudomonads, and four for actinobacteria “first round”) (see Table 5). In a second round, an additional criterion of inclusion was set: to choose the isolates with at least two traits among the assayed parameters. Therefore, another four isolates were selected (50M, 60M, 20P, and 23P). In the last step of the selection, the isolates 45B, 89B, and 114M were excluded because they experienced a viability loss when stored at 4 °C for 3–4 weeks.

The main output of this selection was the choice of 16 isolates, which were analyzed by means of 16S rDNA sequencing (Table 2).

### 3.3. Preliminary Validation in a Growth Chamber

Some selected strains were inoculated as “biofertilizers” during the growth cycle of Saragolla, a durum wheat variety well adapted to a Mediterranean environment; the experiments were performed under controlled conditions in pots. The promising isolates selected in the second step were 16; however, their number was further reduced to eight (12A, 25A, 36M, 40M, 97M, 6P, and 20P), for a better management of the growth chamber. This last selection (best candidates among the best potential strains) was done by using three criteria with a special focus on nutrient-use efficiency: (i) high score for P-mineralization and strains able to perform P-solubilization (also in presence of Fe and Al) (40M, 97M, and 12A); (ii) strains able to produce ammonium and siderophores and to perform nitrification (isolates 6P and 20P); (iii) strains positive to at least four tests (PGBP potentially acting on many traits) (36M, 23P, and 25A).

At the beginning, the viable count of soil was ca. 7 log CFU/g; soil inoculation determined a 1-log increase of the viable counts of the most important groups at the end of the assay; and the pH was 8.2.

**Figure 4:**
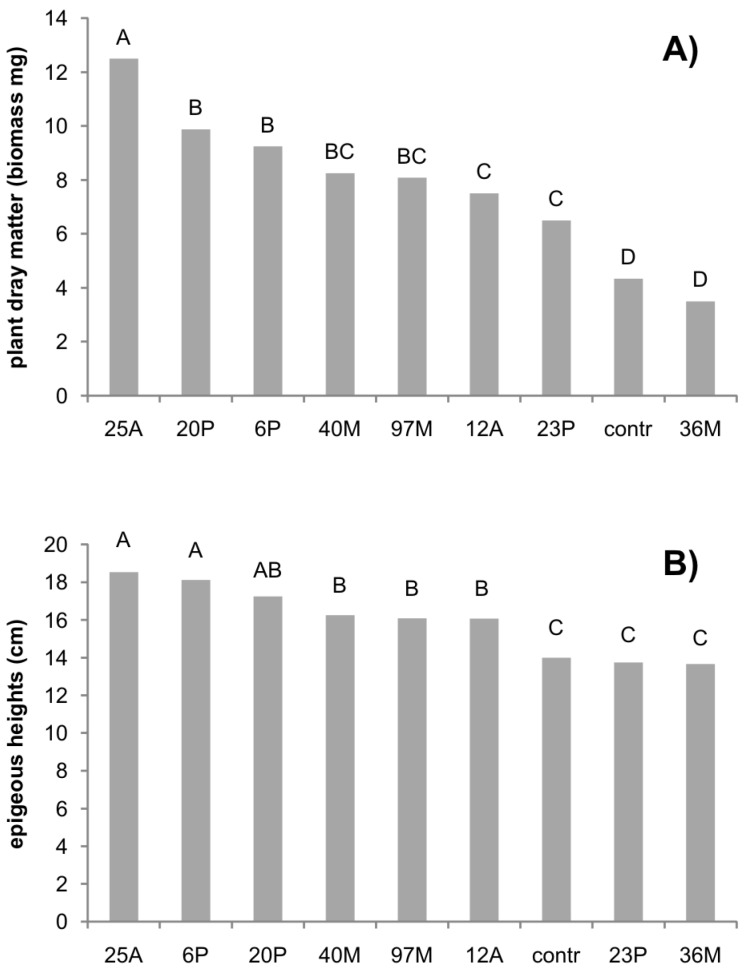
Effect of selected plant-growth-promoting bacteria (PGPB) on durum wheat biomass (**A**) and height (**B**). The letters indicate significant differences (*p* < 0.01)

The results on durum wheat dry biomass and height are reported in Figure 4A,B. Dry matter showed the highest value for the isolate 25A, followed by the isolates 20P and 6P, which all determined at least a 50% increase of biomass if compared to the control. Only one isolate (36M) was not significantly different from the control. The highest height value was found for the isolates 25A and 6P, followed by the isolates 20P, 40M, 97M, and 12A, with a mean increase in height of about 25%.

## 4. Discussion

PGPB are generally used as commercial biofertilizers. A critical issue is that they are allochthonous strains and cannot possess an adaptive capacity; on the other hand, wild strains naturally possess some mechanisms of “adaptive evolution” to win and overcome stressful environmental conditions [19].

To achieve maximum benefits in terms of fertilizer saving and better growth, many critical issues related to PGPB isolation and selection have to be solved: criticisms concern the high number of bacterial species in soil to be analyzed; the choice of the most effective qualitative and quantitative methodologies to select PGPB as biofertilizers, and the statistics adopted for the selection and the validation method in soil of selected autochthonous bacteria.

There are some protocols proposed in the literature for the selection of PGPB; however, in this paper, a new protocol was proposed, based on two main requisites: (i) the use of different levels of selection, with some cut-off points, not set a priori but based on the main statistical distribution of the population, like median and quartiles; (ii) the idea that the selection of microorganisms is a kind of risk-benefit analysis and a compromise between some desired traits and other less desired properties has to be taken into account, as it is not possible to find a super-organism, i.e., a microorganism with all characteristics at their highest score.

The selection of promising microorganisms is a complex process [19], as there is the need to manage a large amount of data coming from many strains; in addition, each strain is characterized by many variables, with different mathematical properties (qualitative or quantitative, discrete or continuous, and binary or multidimensional trends). Therefore, the scenario is a contingency table with many columns and rows.

The main challenge is to reduce the complexity of the spreadsheet but, at the same time, avoid a significant loss of details/information. A second challenge is the definition of inclusion/exclusion criteria to reduce the number of the samples and select the most interesting microorganisms. A possible drawback in this step is the definition of too-restrictive inclusion criteria, thus excluding interesting microorganisms, or to define decision criteria, which are not able to reduce the complexity of the contingency table.

Data clustering and classification can be performed through many techniques and approaches (cluster analysis, principal component analysis (PCA), k-means, and multiple correspondence); each approach has benefits and limitations. However, PCA is the most suitable approach for this research for at least three reasons: (i) it reduces many variables to a smaller number, while losing as little information as possible (reduction of complexity); (ii) it can divide the samples into homogeneous groups (clustering); and (iii) it can offer an idea of the leading variables which play a role in clustering (leading variables).

The first step to reduce the complexity was the definition of the goal, i.e., the selection of promising PGPB faced with a higher nutrient-use efficiency with a focus on P and N. Therefore, some properties were chosen (ammonium production, nitrification, P-solubilization, and P-mineralization); on the other hand, ACC deaminase was not included as a decision criterion. According to Gamalero and Glick [27], it plays a major role in modulating ethylene levels in plant and is involved in the response to biotic and abiotic stress. It is an interesting property, but not directly related to the main goal of nutrient-use efficiency.

One of the main goals of this research was to study the effect of PGPB on N availability (NH_4_^+^ production, nitrification), as nitrogen uptake is linked to plant growth and productivity [28]. N acquisition by roots is strictly dependent on the availability of the source itself, but about 90% of total N is present as SOM (soil organic matter). Therefore, ammonification and then nitrification, carried out by bacteria, are crucial for plant mineral nutrition [28].

Among the four functional clusters analyzed in this paper (pseudomonads, spore-forming bacteria, actinobacteria, and mesophilic bacteria), pseudomonads were the group with the highest capacity, being all the selected isolates able to produce NH_4_^+^ and NO_2_^−^/NO_3_^−^, and this result confirmed some literature reports. For example, Kumar et al. [22] studied 75 isolates of *Pseudomonas* and found that all were NH_4_^+^ producers, although at different levels (weak, moderate, or high). Moreover, *Bacillus* isolates showed a high degree of ammonium production (75%) and nitrification (60%). These results were in agreement with some literature reports [29] and also confirm the potentiality of *Bacillus* and related genera for plant-growth promotion.

Phosphorous, such as nitrogen, is one of the main essential macronutrients required for plants, but 1% or less of the total phosphorus (P) in soil is considered available to plants; [30,31] therefore, phosphate-solubilizing bacteria are of great interest, considering the low P availability in agricultural soils. In fact, they can release soluble P to plants, improving their growth and development [31].

The solubilization of P is considered one the most important traits to select PGPB [32]; however, the classical experiment based on calcium phosphate-solubilization was referred to in the past as being inappropriate [33]. Therefore, after the first selection, the effect on P was confirmed by P-mineralization and P-solubilization of other P-based components (Fe(PO_4_)_3_ and AlPO_4_) [33,34].

In this research, many P-solubilizing bacteria belong to the *Pseudomonas* group, thus confirming the interest toward this group for their action on P [2]. Pseudomonads, along with spore-forming bacteria, were also the cluster with the highest ability to produce siderophores. Bacteria, fungi, and monocotyledonous plants, in response to iron stress, produce and secrete siderophores to sequester iron, in response to an iron stress [35].

An additional criterion to select promising PGPB was the assessment of IAA; bacterial IAA, in fact, together with plant IAA, can regulate some phases of plant development [27,36]. Moreover, this trait could partly offer some details on stress, as IAA can also affect the synthesis of ACC deaminase, thus ethylene content and stress response. In addition, IAA has a key role for plant-growth-promoting activity; apart from the tolerance to stress discussed above, Etesami et al. [37] proposed a global model for the role of bacterial IAA for plants. There are at least three ways or benefits: positive effect on biomass production, enhancement of root elongation, and increase of root exudates. This latter effect is probably the most important, because it, in turn, induces a reduction of soil pH, the release of chelators, and changes in the redox potential, and thus in the solubility of some nutrients. The most important consequence is an increased availability of Fe and P [38,39,40,41]. Due to its strong importance and its significant connection with nutrient availability, IAA was used as a primary criterion to select promising PGPB.

The results on IAA among spore-forming bacteria and pseudomonads were in line with the literature [42], above all, in presence of tryptophan [43,44,45] as the mean value of IAA produced by different classes of bacteria ranges from 10 to 20 to 100 µg/mL. Therefore, the high-producing IAA activity suggests the suitability of some isolates of these clusters to act as plant-growth promoters.

As a result of first and second selection, 16 promising candidates as PGPB bacteria were selected. They all belong to some genera related to the rhizosphere (*Bacillus*, *Pseudomonas*, *Stenotrophomonas*, and *Lysinibacillus*) [33].

The strains were the result of inclusion/exclusion criteria, as some interesting bacteria could be excluded from the selection; however, the main goal of this paper was not to study the microbial diversity related to the rhizosphere of durum wheat or the abundance of some clusters in the soil, but to recover promising candidates for a future validation for durum wheat of Foggia County.

The validation in real conditions is a critical step, as many strains with good traits at lab scale could fail in real conditions. In this paper, a preliminary validation in a growth chamber was proposed.

This kind of approach is only an approximate simulation of real conditions, because a growth chamber is a well-controlled environment; however, it was necessary for a further reduction of the number of promising strains and to choose the best-performers (3 or 4 strains) for a field validation.

As screening and simple indices, two quantitative measures were chosen (effect on height and biomass); although it is well-known that PGPB could exert other effects [27], they are not useful for a screening and quick validation. The main result was the selection of at least three isolates (6P, 20P, and 25A) for a future field validation.

Another possible challenge/topic to be addressed in the future could be the design of a mix (or a cocktail) composed of several promising PGPB selected in this research. Isolates with different promoting traits could collaborate to improve nutrient availability [46,47], as shown for several mixtures of *Bacillus*, *Pseudomonas*, and fungi [48]. Several challenges must be addressed for the design of multi-strain PGPB inoculum, like strain compatibility and the use of microorganisms with different mode of actions, but it is a fact that synthetic microbial multi-strain mixtures show better effects in promoting plant growth and suppressing plant disease compared to individual strains, because the isolates can act on different targets, or a strain could promote the activity of the others by producing stimulating compounds [48].

In conclusion, this paper proposed a statistical flow sheet to select promising PGPB, with two novel traits: (i) the use of cut-off points not set but based on the statistical characteristics of the whole population (median and quartiles); (ii) the idea that it is not possible to find a super organism (i.e., a microorganism with all traits at the highest score), but a microorganism with some interesting traits faced to specific goals (in this paper, use efficiency of N and P).

In addition, the selection was faced to a crop (durum wheat) in a well-defined environment (Foggia County, Southern Italy), as each niche harbors a different microbiota and is a source of promising PGPB for that environment.

The results of this paper are the background for future efforts and research; first, the promising strains should be validated in real conditions (field), and their technological robustness at the industrial level should be assessed for an effective production of commercial biofertilizer. Moreover, the statistical flow sheet hereby proposed could be validated for other environments and implemented with other traits (for example, ACC deaminase, enzymes, etc.), to contribute to international guidelines for the identification and selection of PGPB.

## Figures and Tables

**Table 1:** Growth-chamber cycle and physical and chemical properties of the soil used for the experiment.

Growth-Chamber Cycle					
Days	Photoperiod (Time 24 h)		Temperature Day Night		Humidity% Day Night
1–9			6 °C	6 °C	65%
10–19	7.00	17.00	10 °C	8 °C	65%
20–29	6.00	18.30	15 °C	10 °C	65%
30–39	6.00	20.00	18 °C	12 °C	65%
**Soil Properties**					
Sand		%		23.1	
Silt		%		56.3	
Clay		%		20.5	
Organic carbon †		g/kg		1.6	
Available P ‡		mg/kg		54.9	
K assimilable (K_2_O)		mg/kg		260	
Total N ^§^		%		1.1	
NO_3_^−^		mg/kg		15.5	
Soil pH				8.2	

† Walkley-black method, ‡ Olsen method, ^§^ Kjeldhal method.

**Table 2:** Identification and characteristics of the isolates selected after the second step of the research (quantitative assays). Cat., catalase; ox., oxidase; P-solubilization: assays performed in presence of Ca_3_(PO_4_)_2_, AlPO_4_, and Fe(PO_4_)_3._

Isolate	GenBank Accession Numbers	Identification	Cat.	Ox.	Urease	Motility	Sider. Production	NH_4_^+^ Production	P-Solubilization (halo, mm)			P- Mineralization (µM)	IAA µg/mL)	Nitrifi-cation (µM)
									Ca_3_(PO_4_)_2_	AlPO_4_	Fe(PO_4_)_3_			
**36M**	*MG515459*	*Bacillus* spp.	−	+	−	+	+	+	1	3	2	4.90 ± 0.85	0.98 ± 0.14	0.99 ± 0.02
**40M**	*MG515460*	*Bacillus* spp.	+	+	−	+	+	+	1	2	0	4.79 ± 0.24	/	/ ^§^
**50M**	*MG515469*	*Bacillus* spp.	+	+	−	+	+	+	1	0	1	2.15 ± 0.41	0.98 ± 0.28	1.99 ± 0.08
**54M**	*MG515466*	*Bacillus simplex Brevibacterium frigoritolerans*	+	−	−	−	+	−	0	1	0	/	/	17.88 ± 0.01
**58M**	*MG515457*	*B. simplex* *Br. frigoritolerans*	+	−	−	−	+	−	0	1	0	/	/	18.41 ± 0.21
**60M**	*MG515467*	*B. simplex* *Br. frigoritolerans*	+	−	−	−	+	−	0	2	0	/	/	17.56 ± 0.01
**97M**	*MG515461*	*Bacillus* spp.	+	+	−	+	+	+	1	1	1	8.90 ± 0.44	/	/
**3B**	*MG515470*	*Bacillus* spp.	−	+	−	+	−	+	3	1	2	2.89 ± 0.15	2.96 ± 0.07	0.40 ± 0.17
**19B**	*MG515471*	*Bacillus* spp.	+	+	−	+	−	+	4	1	0	1.48 ± 0.07	5.72 ± 0.29	7.18 ± 0.15
**6P**	*MG515464*	*Stenotrophomonas tumulicola*	+	−	−	+	+	+	0	2	0	/	/	17.60 ± 0.48
**20P**	*MG515465*	*Stenotrophomonas* spp.	+	−	−	−	+	+	2	5	0	/	/	15.82 ± 0.46
**23P**	*MG515462*	*Pseudomonas migulae*	+	+	−	−	+	+	4	2	2	2.17 ± 0.11	3.65 ± 0.42	/
**10A**	*MG515468*	*Lysinibacillus* spp.	−	−	−	−	−	−	0	0	2	/	/	18.69 ± 0.34
**12A**	*MG515472*	*Bacillus* spp.	+	+	−	−	−	+	2	6	3	8.01 ± 0.33	5.82 ± 0.18	/
**25A**	*MG515463*	*Bacillus* spp.	+	+	−	−	+	+	3	2	2	3.96 ± 0.20	/	/
**145A**	*MG515458*	*Bacillus endophyticus*	+	+	−	−	−	+	0	1	0	/	140.31 ± 7.00	/

^§^ Not assessed.

**Table 3:** Screening on some phenotypic tests on the isolates. Percentages of isolates positive to the test. The motility was evaluated at the optimal temperatures (30 °C for mesophilic and spore-forming bacteria, 22 and 25 °C for actinobacteria and pseudomonads).

Bacteria	Number of Isolates	Gram Positive	Motility	Catalase Production	Oxidase Production	Urease
*Bacillus* spp.	96	100	11	96	39	11
Mesophilic Bacteria	133	89	9	90	30	6
*Pseudomonas* spp.	65	0	30	100	80	1
Actinobacteria	180	87	6	97	52	10

**Table 4:** Quantitative assays: median and quartiles.

	Phosphate-Mineralization (µM)	Indole Acetic Acid (µg/mL)	Nitrification (µM)
**Mesophilic Bacteria**			
Median	1.81	18.86	13.13
1 quartile	1.12	5.23	1.48
3 quartiles	2.61	38.91	17.51
**Spore-Forming Bacteria**			
Median	1.49	5.33	1.62
1 quartile	0.94	3.75	0.78
3 quartiles	1.79	9.09	1.97
**Pseudomonads**			
Median	0.91	2.27	15.83
1 quartile	0	1.38	8.20
3 quartiles	0	3.65	17.32
**Actinobacteria**			
Median	0.49	7.21	2.44
1 quartile	0.19	4.25	0.91
3 quartiles	1.61	26.38	8.16

**Table 5:** Selected isolates. Codes and criteria for the choice. P, P-mineralization; IAA, indole acetic acid production; nit, nitrification.

First Round				
Isolates	P	IAA	Nit	Why
36M	3	1	1	Isolates with the highest level of P among mesophilic bacteria
40M	3	0	0	
97M	3	0	0	
54M	0	0	3	Isolates with the highest level of Nit among mesophilic bacteria
58M	0	0	3	
114M †	0	3	0	Highest level of IAA among mesophilic bacteria
3B	3	1	1	Isolate with the highest level of P among spore-forming bacteria
19B	1	2	3	Isolate with the highest level of Nit among spore-forming bacteria
45B †	0	3	0	Highest level of IAA among mesophilic bacteria
89B †	0	3	0	
6P	0	0	3	Isolate with the highest level of Nit among pseudomonads
10A	0	0	3	Isolate with the highest level of Nit among actinobacteria
12A	3	1	0	Isolates with the highest level of P among actinobacteria
25A	3	0	0	
145A	0	3	0	Isolate with the highest level of IAA among actinobacteria
**Second Round**				
50M	2	1	1	3 properties and high resistance
60M	2	1	1	3 properties and high viability and resistance
20P	0	1	2	2 properties and high viability and resistance
23P	3	3	0	2 properties at levels > 3 quartiles

† 114M, 45B, and 89B: these isolates showed a low viability; thus, they were excluded and not used for the last step of the research (identification and growth-chamber assay).

## Supplementary Materials

The following are available online at https://www.mdpi.com/2076-2607/7/11/541/s1. Figure S1: Soil samples around the roots. Figure S2: Principal component analysis run on pseudomonads. Figure S3: Principal component analysis run on actinobacteria. Table S1: Second selection. Qualitative codes based on medians and quartiles. P, mineralization of P; IAA, production of indole acetic acid; nitr, nitrification. Table S2-A: One-way ANOVA (homogeneous group approach) on P-mineralization by mesophilic bacteria, S2-B: One-way ANOVA (homogeneous group approach) on P-mineralization by spore-forming bacteria, S2-C: One-way ANOVA (homogeneous group approach) on P-mineralization by pseudomonads, and S2-D: One-way ANOVA (homogeneous group approach) on P-mineralization by actinobacteria.
