# Supplementary material for: Isolation, Screening, and Characterization of Plant-Growth-Promoting Bacteria from Durum Wheat Rhizosphere to Improve N and P Nutrient Use Efficiency
Source: Microorganisms. 2019 Nov 8;7(11):541. doi: 10.3390/microorganisms7110541 (PMC6920805; doi:10.3390/microorganisms7110541)
Supplement: Supplementary file 1 [file microorganisms-07-00541-s001.pdf]

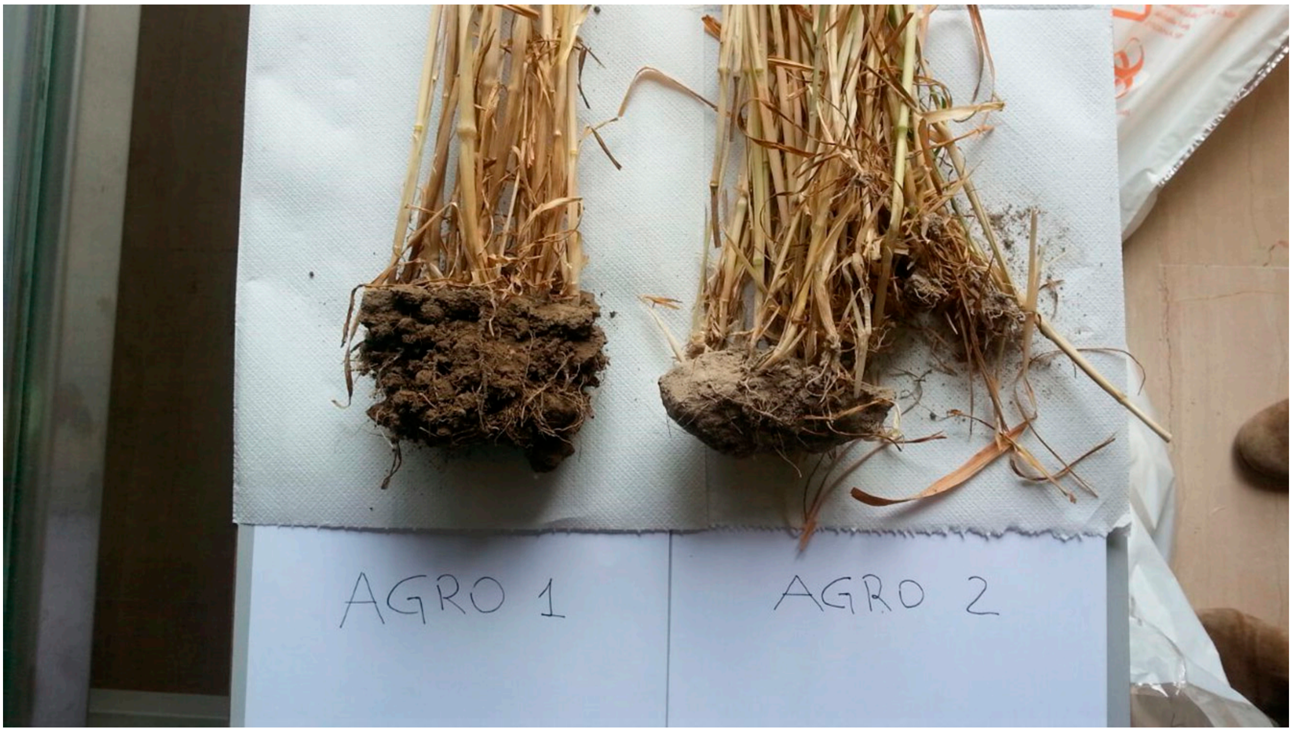

Figure S1: Soil samples around the roots

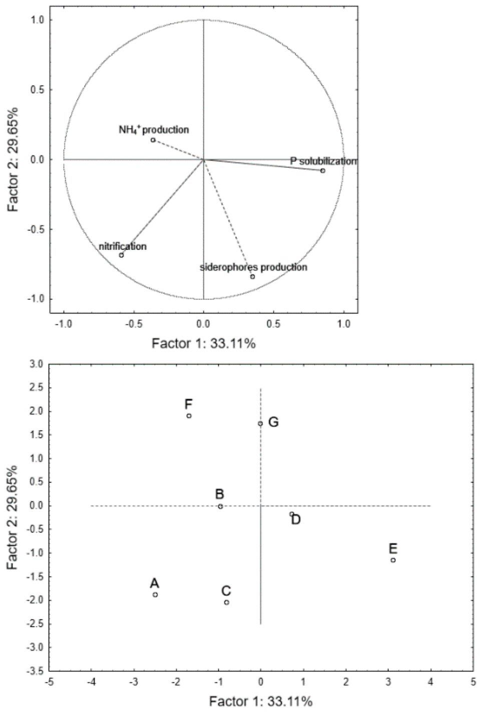

	Isolates	1	2	3	4
**A**	6P, 15P, 54P, 56P	+	+	-	+
**B**	12P, 14P, 32P, 47P, 53P	+	-	-	+
**C**	19P, 20P, 30P	+	+	+	+
**D**	7P, 8P, 10P, 34P, 35P, 36P, 37P, 38P, 39P, 43P, 44P, 45P, 46P, 48P, 49P, 50P, 51P, 52P, 55P, 57P, 59P, 62P	+	-	+	+
**E**	42P	-	-	+	+
**F**	26P, 24P, 27P, 29P, 56P	+	-	-	-
**G**	3P, 4P, 60P, 61P, 63P, 64P	+	-	+	-

1, NH_4^+ production; 2, Nitrification; 3, P-solubilization; 4, siderophore production

Figure S2: Principal Component Analysis run on pseudomonads.

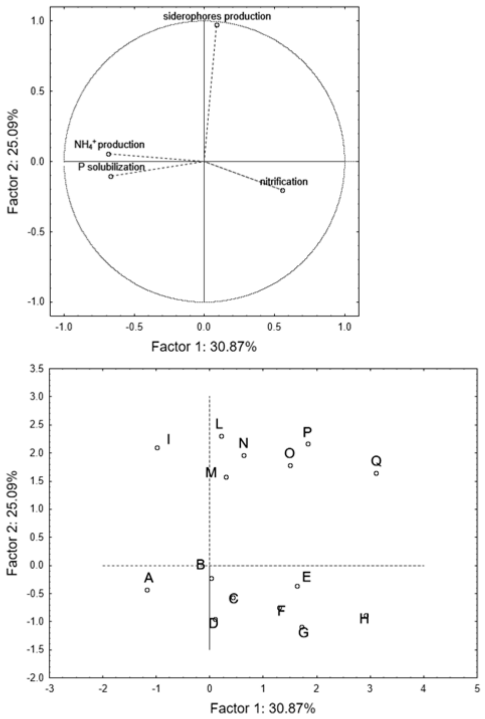

	Isolates	1	2	3	4
**A**	3A, 6A, 12A, 14A, 17A, 18A, 21A, 23A, 28A, 31A, 48A, 49A, 50A, 56A, 72A, 80A, 84A, 85A, 86A, 90A, 91A, 92A, 93A, 95A, 98A, 105A, 114A, 125A, 130A, 131A, 133A, 141A, 146A, 147A, 149A, 166A, 176A, 177A	+	-	+	-
**B**	7A, 13A, 16A, 22A, 32A, 39A, 2A, 5A, 40A, 44A, 51A, 54A, 55A, 60A, 69A, 70A, 74A, 75A, 87A, 99A, 101A, 102A, 111A, 119A, 143A, 145A, 153A, 157A, 159A, 160A, 164A, 174A	+	-	-	-
**C**	122A, 123A, 132A, 151A, 165A	-	-	+	-
**D**	62A, 71A, 100A, 117A, 156A, 158A	+	+	+	-
**E**	57A, 79A, 113A, 126A, 144A, 170A, 173A, 175A	-	-	-	-
**F**	42A, 43A, 61A, 65A, 78A, 97A, 115A, 138A	+	+	-	-
**G**	83A	-	+	+	-
**H**	10A, 116A, 136A, 142A	-	+	-	-
**I**	25A, 26A, 82A, 104A, 108A, 124A, 137A	+	-	+	+
**J**	8A, 24A, 34A, 38A, 47A, 107A, 129A, 179A	+	-	-	+
**K**	1A, 103A	+	+	+	+
**L**	109A	-	-	+	+
**M**	112A	+	+	-	+
**N**	110A, 167A	-	-	-	+
**O**	148A	-	+	-	+

1, NH_4^+ production; 2, Nitrification; 3, P-solubilization; 4, siderophore production

Figure S3: Principal Component Analysis run on actinobacteria.

Table S1: Second selection. Qualitative codes based on medians and quartiles. P, mineralization of P; IAA, production of indole acetic acid; nitr, nitrification.

Actinobacteria

	P	IAA	nitr
1A	0	0	1
3A	1	0	0
10A	0	0	3
12A	3	1	0
13 A	0	2	0
17A	1	1	0
18 A	0	1	0
20 A	0	2	0
23A	3	0	0
25A	3	0	0
26 A	0	2	0
28 A	0	1	0
31A	1	1	0
32 A	0	3	0
37 A	0	1	0
38 A	0	1	0
42A	0	0	3
47 A	0	1	0
48 A	2	1	0
49 A	0	1	0
50 A	0	1	0
51 A	0	1	0
54 A	0	1	0
55 A	0	1	0
56 A	0	1	0
65 A	0	1	0
71A	2	1	0
72 A	0	1	0
74 A	0	1	0
75 A	0	1	0
78 A	0	1	1
80A	1	0	0
84 A	0	1	0
85 A	0	1	0
90A	3	1	0
92 A	0	1	0
93A	1	0	0
94 A	0	1	0
95 A	0	3	0
97 A	0	3	0
98 A	0	1	0
100 A	0	2	2
103 A	0	1	1
104A	2	1	0
105 A	0	2	0
107 A	0	3	0
108 A	0	2	0
109 A	0	3	0
110 A	0	2	0
111 A	0	1	0
112 A	0	3	3
115A	0	0	1
122A	1	2	0
124 A	0	1	0
125 A	0	2	0
129 A	0	1	0
133 A	0	2	0
135 A	0	1	0
136 A	0	1	2
138 A	0	2	0
139 A	0	2	0
140 A	0	2	0
141 A	0	2	0
143 A	0	2	0
144 A	0	3	0
145 A	0	3	0
147 A	0	1	0
148 A	0	2	0
149A	1	0	0
151 A	2	1	0
153 A	0	3	0
155 A	0	2	0
156 A	0	3	1
158 A	0	3	0
159 A	0	3	0
160 A	0	3	0
164 A	0	3	0
165 A	1	1	0
166 A	0	1	0
167 A	0	3	0
168 A	0	2	0
170 A	0	2	0
171 A	0	3	0
172 A	0	3	0
175 A	0	3	0
176 A	0	3	0

Mesophiles

	P	IAA	nitr.
4M	3	0	0
11M	1	0	0
16M	3	0	0
17M	2	0	0
18M	1	0	0
19M	3	0	0
20M	3	0	0
21M	1	0	0
22M	2	0	0
23M	2	0	1
26M	1	0	0
27M	2	1	0
29M	1	0	0
30M	1	0	0
32M	0	0	1
35M	3	0	0
36M	3	1	1
38M	0	0	2
40M	3	0	0
41M	2	0	0
43M	1	0	0
45M	0	0	2
48M	3	0	0
50M	2	1	1
54M	0	0	3
58M	0	0	3
59M	1	0	0
60M	0	0	3
61M	1	0	1
62M	0	1	0
63M	2	0	0
67M	2	0	0
68M	0	1	0
72M	3	0	0
73M	0	1	0
74M	2	2	0
75M	0	1	0
77M	1	1	1
78M	0	1	0
79M	1	0	0
84M	1	1	0
86M	2	0	0
87M	1	1	0
91M	0	2	0
92 M	0	1	0
93M	0	0	2
96 M	0	1	0
97M	3	0	0
100M	1	0	0
105M	0	2	0
106M	0	2	0
109M	1	1	0
111M	0	1	0
112M	0	3	0
113M	1	2	0
114M	0	3	0
115M	1	3	0
116M	0	3	0
117M	1	3	0
118M	3	0	0
119M	1	2	0
120M	1	2	0
121M	0	1	0
122M	0	1	0
123M	0	2	0
124M	0	2	0
125M	2	3	0
127M	1	2	0
128M	0	3	0
129M	0	3	0
130M	0	3	0
131M	1	1	0
133M	1	0	0

Pseudomonads

	P	IAA	nitr.
6P	0	0	3
7P	0	3	0
10P	0	3	0
11P	0	2	0
13P	0	3	0
14P	0	3	0
15P	0	0	1
16P	0	1	0
19P	0	1	1
20P	0	1	2
23P	3	3	0
26P	0	1	0
30P	1	0	1
37P	0	2	0
47P	0	3	0
48P	0	1	0
49P	0	1	0
50P	0	1	0
51P	0	1	0
52P	0	1	0
54P	0	1	3
56P	0	0	2
58P	0	1	0
62P	2	0	0

Spore-forming bacteria

	P	IAA	nitr.
**2 B**	0	1	0
**3B**	3	1	1
**4B**	1	3	0
**5 B**	0	3	0
**6 B**	0	2	0
**7 B**	0	1	0
**8 B**	0	1	1
**9 B**	0	1	0
**10 B**	0	1	0
**12 B**	0	1	0
**13 B**	0	1	0
**15 B**	0	1	2
**16B**	3	2	0
**17 B**	0	1	0
**18 B**	0	1	0
**19B**	1	2	3
**20 B**	0	2	0
**21 B**	0	1	0
**22B**	1	2	1
**23 B**	0	1	0
**24 B**	0	2	0
**26 B**	0	1	3
**27B**	2	1	0
**28B**	2	0	0
**29B**	1	0	0
**30 B**	0	1	3
**31 B**	0	2	0
**32 B**	0	1	0
**33 B**	1	1	0
**34B**	0	2	3
**35B**	3	2	0
**36 B**	0	1	0
**37B**	3	1	0
**38 B**	0	2	0
**39 B**	0	1	0
**40 B**	0	1	0
**42 B**	0	3	0
**43 B**	0	3	0
**45 B**	0	3	0
**46 B**	0	2	0
**47 B**	0	2	0
**48 B**	0	3	0
**49B**	1	3	0
**50B**	2	0	0
**51 B**	0	1	0
**53 B**	0	1	0
**54 B**	0	3	0
**55 B**	0	1	0
**56B**	2	0	0
**57 B**	0	1	0
**58B**	3	3	0
**60B**	0	3	2
**61B**	1	1	1
**62 B**	0	3	0
**63 B**	0	2	0
**66 B**	0	1	0
**67 B**	0	1	2
**68 B**	0	1	0
**69 B**	0	1	1
**70 B**	0	2	0
**71 B**	0	3	0
**72 B**	0	1	0
**73 B**	0	1	0
**74 B**	0	3	0
**75 B**	0	3	0
**77B**	0	0	3
**79 B**	0	1	0
**80 B**	0	2	1
**82 B**	0	1	0
**83 B**	0	3	1
**84B**	1	1	0
**86 B**	0	3	0
**87 B**	0	3	0
**88 B**	0	2	0
**89 B**	0	3	0
**90 B**	0	3	0
**91 B**	0	1	0
**93 B**	0	3	1
**94 B**	0	2	0
**95 B**	0	1	0
**96 B**	1	3	0

Table S2-A: One-way ANOVA (homogeneous group approach) on P-mineralization by mesophilic bacteria.

Isolate	P-min. (µM)	Homogeneous group		
		I	II	III
**4M**	3.14	■		
**48M**	3.23	■		
**20M**	3.28	■		
**19M**	3.35	■		
**118M**	3.67	■		
**16M**	3.83	■		
**35M**	4.30	■		
**40M**	4.79	■	■	
**36M**	4.90	■	■	
**97M**	8.90			■

Table S2-B: One-way ANOVA (homogeneous group approach) on P-mineralization by spore-forming bacteria.

Isolate	P-min. (µM)	Homogeneous group				
		I	II	III	IV	V
**96B**	0.17	■				
**29B**	0.54	■	■			
**61B**	0.55	■	■			
**49B**	0.66		■			
**33B**	0.81		■			
**84B**	1.33			■		
**22B**	1.34			■		
**4B**	1.38			■		
**19B**	1.48			■		
**50B**	1.50			■		
**28B**	1.54			■		
**56B**	1.64			■		
**27B**	1.78			■		
**37B**	1.79			■		
**35B**	2.35				■	
**16B**	2.36				■	
**58B**	2.56				■	■
**3B**	2.89					■

Table S2-C: One-way ANOVA (homogeneous group approach) on P-mineralization by pseudomonads.

Isolate	P-min. (µM)	Homogeneous group
**30P**	0.64	■
**62P**	0.91	■
**23P**	2.17	■

Table S2-D: One-way ANOVA (homogeneous group approach) on P-mineralization by actinobacteria.

Isolate	P-min. (μM)	Homogeneous group
**93A**	0.13	
**17A**	0.14	
**31A**	0.14	
**122A**	0.16	
**80A**	0.20	
**165A**	0.35	
**3A**	0.47	
**149A**	0.49	
**104A**	0.50	
**151A**	0.62	
**71A**	1.27	
**48A**	1.50	
**90A**	1.94	
**23A**	2.99	
**25A**	3.96	
**12A**	8.01	
